# Buffalo Academy of Medicine

**Published:** 1898-10

**Authors:** Thomas F. Dwyer


### Society Proceedings.

### BUFFALO ACADEMY OF MEDICINE.

REPORTED BY THOMAS F. DWYER, M. D., Secretary.

THE quarterly meeting was held at the Academy parlors, Palace Arcade, Tuesday evening, September 13, 1898.

In the absence of the president, the chairman of the section of medicine, Dr. William G. Ring, presided and called the meeting to order at 8.45 p. m.

The minutes of the last quarterly meeting, and the special meeting of July 11, 1898, were read and approved.

The council recommended the election for resident membership of the following applicants: Drs. Albert E. Woehnert, Charles P. Knowles and Norman L. Burnham. They were separately balloted for and duly elected Fellows of the Academy.

The proposed amendments to the by-laws, notice of which had been given in writing at the last stated meeting and had been announced on the call for this meeting, were then read by the secretary.

Amendment I.—" That names of members who are six months or more in arrears for dues, and the amounts due be posted in a conspicuous place in the Academy rooms until same are paid."

It was moved and seconded that the amendment be carried as read. This motion was lost by a vote of 24 to 11.

Amendment II.—" That Article XIII., Section 3, of the by-laws be amended to read: Any Fellow failing to pay his dues for two years shall forfeit his fellowship."

This amendment was unanimously carried.

It was moved and seconded that a committee be appointed to revise the constitution and by-laws. It was moved as an amendment that the secretary be empowered to have slips printed, giving the changes made since the last revision of the constitution and by-laws and sent to the members of the Academy. Motion as amended was carried.

The program of the evening, under auspices of the section of medicine, consisted of the following: Chronic joint affections, commonly called rheumatism, Charles G. Stockton, M. D.; Rheumatism of childhood, Benjamin G. Long, M. D. The first paper was discussed by Drs. Mynter, Rochester, Blaauw and Van Peyma, and the second by Drs. Snow, Dwyer, Mynter, Stockton and Rochester. The attendance at the meeting was forty-one.

Adjourned at 11 p. m.

